# Huoxue Jiedu Huayu Formula Alleviates Cell Pyroptosis in Contralateral Kidneys of 6-Month-Old UUO Rats through the NLRP3/Caspase-1/IL-1*β* Pathway

**DOI:** 10.1155/2021/5533911

**Published:** 2021-07-12

**Authors:** Xuelian Ma, Panpan Qiang, Gege Chen, Zheng Wang, Xiangting Wang, Qingyou Xu

**Affiliations:** ^1^Hebei Key Laboratory of Integrative Medicine on Liver-Kidney Patterns, Hebei University of Chinese Medicine, Shijiazhuang, China; ^2^Department of Internal Medicine, Hebei University of Chinese Medicine, Shijiazhuang, China; ^3^Graduate School, Hebei University of Chinese Medicine, Shijiazhuang, China

## Abstract

**Objectives:**

To study the protective effects and mechanisms of Huoxue Jiedu Huayu formula on cell pyroptosis through the NLRP3/caspase-1/IL-1*β* pathway in contralateral kidneys in 6-month-old unilateral ureteral obstruction (UUO) rats.

**Methods:**

Wistar rats were randomly assigned to 5 groups: a Sham group, a unilateral nephrectomy group (UNX group), a UUO group, a UUO treated with spironolactone group (Spi group), and a UUO treated with Huoxue Jiedu Huayu formula group (HJHF group). After 6 months of oral drug intervention, blood and contralateral kidneys were collected for research.

**Results:**

The morphology and function of the contralateral kidneys were essentially normal after unilateral nephrectomy. HJHF obviously decreased serum creatinine, urea, and inflammatory lesions and depressed cell pyroptosis based on the NLRP3/caspase-1/IL-1*β* pathway. Moreover, spironolactone, a mineralocorticoid receptor (MR) blocker, suppressed cell pyroptosis through SGK-1 and NF-кB.

**Conclusion:**

HJHF and spirolactone inhibited excessive activation of MR and then reduced cell pyroptosis, which was dependent on the NLRP3/caspase-1/IL-1*β* pathway, to protect the contralateral kidneys of 6-month-old UUO rats.

## 1. Introduction

Obstructive nephropathy accounts for 15.59% of chronic kidney disease (CKD) in China [1]. Obstructive nephropathy resulting from unilateral kidney or ureter obstruction is closely related to the obstruction of the contralateral kidney [2–5]. Protecting the contralateral kidney from obstruction may prevent the onset and progression of obstructive nephropathy. However, the mechanisms of contralateral kidney injury caused by unilateral kidney obstruction still need to be researched.

Treatment of CKD with traditional Chinese medicine (TCM) is widely recognized in China [6–8]. Although the pathological factors are complex, the pathogenesis of CKD, according to the theory of TCM, can be summarized as debility, stasis, and toxicity. In the clinic, Huoxue Jiedu Huayu formula (HJHF) can improve kidney function, reduce urinary protein, and delay the occurrence of kidney failure due to chronic obstructive nephropathy. However, the underlying molecular mechanisms of treatment are not well understood.

Aldosterone promotes the development of obstructive nephropathy and CKD through inflammatory injury [9–11]. High plasma aldosterone levels are often associated with proteinuria and glomerulosclerosis. Mineralocorticoid receptor (MR) plays an important role in the inflammatory injury of aldosterone [12–14]. Pyroptosis is a type of cell death that can induce severe inflammation [15]. Nucleotide-binding oligomerization domain-like pyrin domain containing protein 3 (NLRP3) is one of the inflammasomes that induces pyroptosis and can be activated by aldosterone [16]. NLRP3/caspase-1/IL-1*β* is the classic pathway of pyroptosis [17, 18]. Whether aldosterone/MR-related pyroptosis promotes the development of obstructive nephropathy remains to be studied.

In our previous studies, we observed cell pyroptosis and the inhibitory effect of HJHF on pyroptosis with mesangial cells in the contralateral kidneys of short-term (10-day) UUO rats [19, 20]. For this study, we used 6-month-old unilateral ureteral obstruction (UUO) rats to model the process of chronic obstructive nephropathy to observe the effect of HJHF on contralateral kidney injury and then explored the mechanisms of aldosterone-induced pyroptosis on the basis of the NLRP3/caspase-1/IL-1*β* pathway.

## 2. Materials and Methods

### 2.1. Animal Grouping and Model Preparation

Seventy-five male Wistar rats (6∼8 weeks old, 190∼210 g, SPF grade) were supplied by the Experimental Animal Center of Hebei Province (license number: SCXK (Ji) 2013-1-003). After a week of adaptive feeding at 25°C, all rats were randomly assigned to five groups (15 rats per group): a Sham group, a unilateral nephrectomy group (UNX group), a UUO group, a UUO treated with spironolactone group (Spi group), and a UUO treated with Huoxue Jiedu Huayu formula group (HJHF group). All rats underwent surgery under 3% isoflurane anesthesia and asepsis. Rats from the UUO, Spi, and HJHF groups were subjected to ligation of the left ureters following routine steps. The left kidneys from the Sham and UNX groups were exposed and resected following the protocol described in a previous study [21]. The processes for the rat experiments were authorized by the Ethics Committee of Hebei University of Chinese Medicine.

### 2.2. Drug Administration

The rats from the Sham, UUO, and UNX groups were fed tap water and a routine diet. Every day, all rats from the Spi and HJHF groups were orally administered water with dissolved spironolactone (20 mg/kg) and HJHF herbs (1.92 g/kg), respectively, for 6 months after UUO surgery. Spironolactone was produced by Minsheng Pharmaceutical Co. (Hangzhou, China). Granules extracted from HJHF herbs were purchased from Yifang Pharmaceutical Co. (Foshan, China). The HJHF consisted of 5 Chinese Materia Medica: Huangqin (*Radix Scutellariae*), Huangqi (*Radix Astragali*), Chishao (*Radix Paeoniae Rubra*), Biejia (*Carapax Trionycis*), and Dilong (*Lumbricus*), with a mass ratio of 18 : 40 : 20 : 7 : 10.

### 2.3. General Condition Examination and Sample Collection

The systolic blood pressure of rats in each group was measured by using an animal noninvasive blood pressure analysis system (BP-2000, Visitech Systems Co., USA) every month after UUO. Next, all rats were euthanized and weighed after 6 months of UUO. Meanwhile, the contralateral kidneys (right kidneys) and blood from the femoral vein were harvested for study. The contralateral kidneys were also weighed to calculate the kidney- to-body weight ratio.

### 2.4. Kidney Function and Serum Aldosterone Analysis

Serum creatinine and urea were detected using an automatic biochemical analyzer (HEMIX-180, Sysmex Co., Japan) according to the steps of the creatinine kit (picric acid method, lot: AUZ3562, Bio-Rad, USA) and the blood urea kit (urease glutamate dehydrogenase method, lot: AUZ3611, Bio-Rad, USA), respectively. Serum aldosterone was tested by enzyme-linked immunosorbent assay (ELISA) following the instructions of the ELISA aldosterone detection kit (double antibody sandwich method, lot: GR298352-5, Abcam, UK).

### 2.5. Kidney Histopathology Analysis

The kidneys were fixed with 4% paraformaldehyde solution, embedded in paraffin, and then cut into 5 *μ*m thick slices for hematoxylin-eosin (HE) staining, Masson staining, and Sirius red staining. The histological structure and collagen deposition of the kidneys were observed and photographed by using a microscope (BX53, Olympus Co., Japan).

### 2.6. TdT-Mediated dUTP Nick End Labeling (TUNEL)

Paraffin sections of kidneys were detected by an in situ cell death detection kit, POD, according to the manufacturer's instructions (lot: 11422200, Roche, Switzerland). The nuclei were counterstained with hematoxylin. Quantitative method was used for counting TUNEL-positive cells: sections from 4 rat kidneys were randomly selected for TUNEL detection in each group. Five fields of each section were randomly selected under the microscope at 400x magnification, and the number of positive cells was recorded. Finally, the average number of positive cells per field in each group was calculated.

### 2.7. Immunohistochemistry Analysis

The dewaxed sections were incubated in 3% H_2_O_2_ solution to eliminate endogenous peroxidase activity and were then pressurized in citrate buffer solution (0.01 mol/L, pH = 6) to expose the antigen. Next, the sections were dropped in 10% goat serum and incubated with the NR3C2 antibody (1 : 100, lot: 21854-1-AP, Proteintech, USA). After that, the sections were incubated with biotinylated secondary antibody and HRP-conjugated streptavidin in turn. Finally, the sections were positive for diaminobenzidine (DAB) and photographed by using a microscope (BX53, Olympus Co., Japan).

### 2.8. Immunofluorescence Test

After dehydration with 30% sucrose and embedding with optimal cutting temperature compound (OCT), the kidney tissue was sliced by a freezing microtome with a thickness of 7 *μ*m. Next, kidney sections were incubated with rabbit or mouse antibodies against CD68 (1 : 200, lot: ab955, Abcam), NLRP3 (1 : 100, lot: F1716, Eterlife, UK), caspase-1 (1 : 200, lot: 00046167, Proteintech, USA), and IL-1*β* (1 : 200, lot: NB60-633, Novus, USA). Then, the sections were successively incubated with fluorescently labeled secondary antibodies and 4,6-diamidino-2-phenylindole (DAPI). Finally, the sections were observed by using fluorescence microscopy.

### 2.9. Western Blot Assay

Kidney tissue protein was extracted in lysis buffer and then separated by sodium dodecyl sulfate-polyacrylamide gel electrophoresis (SDS-PAGE). Next, the protein was transferred to polyvinylidene fluoride (PVDF) membranes in a semidry film transfer instrument. The PVDF membranes were incubated with primary antibodies, including antiserum and glucocorticoid-induced protein kinases 1 (anti-SGK-1) (1 : 500, lot: GR197317-12, Abcam, UK), anti-nuclear factor-кB (anti-NF-кB (p65)) (1 : 1000, 181905, Servicebio, China), anti-caspase-1 (1 : 1000, lot: 00046167, Proteintech, USA), anti-NLRP3 (1 : 500, lot: F1716, Eterlife, UK), and anti-IL-1*β* (1 : 1000, lot: NB60-633, Novus, USA). Then, the PVDF membranes were incubated with infrared fluorescence-conjugated secondary antibodies. Using glyceraldehyde-3-phosphate dehydrogenase (GAPDH) as a reference, proteins on the membranes were quantitatively analyzed using an infrared imaging system (Odyssey, LI-COR, USA).

### 2.10. Statistical Analysis

GraphPad Prism 7 (GraphPad Software Inc., USA) was used for data analysis. The data are represented by the mean ± standard deviation. One-way ANOVA and Student–Newman–Keuls Q tests were applied to compare differences between groups. *P* *<* 0.05 was considered statistically significant.

## 3. Results

### 3.1. Observations of 6-Month-Old Unilateral Nephrectomy Rats

**Figure 1:**
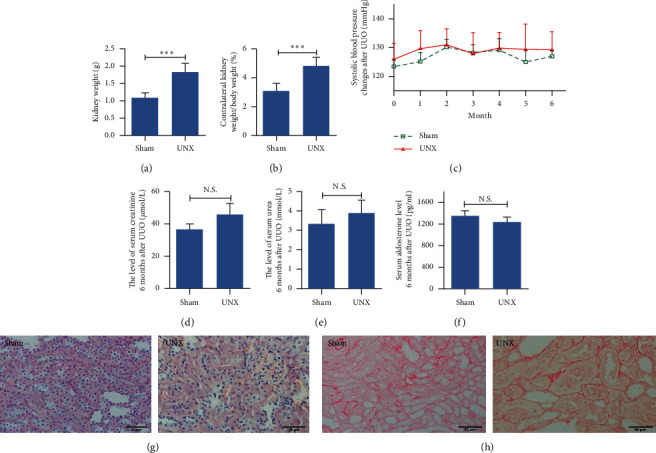
No significant injury was observed in the contralateral kidneys of 6-month-old unilateral nephrectomy rats. (a, b) Compared with the Sham group, the kidney weight and the ratio of kidney to body weight were significantly increased in the UNX group. ^*∗∗∗*^*p* < 0.001. (c–e) Systolic blood pressure and the levels of serum creatinine and urea and serum aldosterone were not notably different in the UNX and Sham groups. (g, h) Comparing UXN with the Sham group, renal histology and collagen deposition were not remarkably changed by HE and Sirius red staining except for some tubules and glomeruli hypertrophy.

The histogram in Figure 1 shows that the kidney weight and the ratio of the contralateral kidneys (right kidneys) to body weight were significantly increased in the UNX group. The glomeruli and kidney tubules were normal, and there were small amount of collagens in the basement membranes of the kidney tubules in both groups. In addition, we also observed glomerular and kidney tubule hypertrophy in the UNX group. Systolic blood pressure, serum creatinine and urea, and serum aldosterone were not notably different between the two groups. Thus, high pressure and perfusion from unilateral nephrectomy did not result in severe damage to the contralateral kidney (Figure 1).

### 3.2. Improvement of Kidney Function after Treatment with Huoxue Jiedu Huayu Formula and Spironolactone

**Figure 2:**
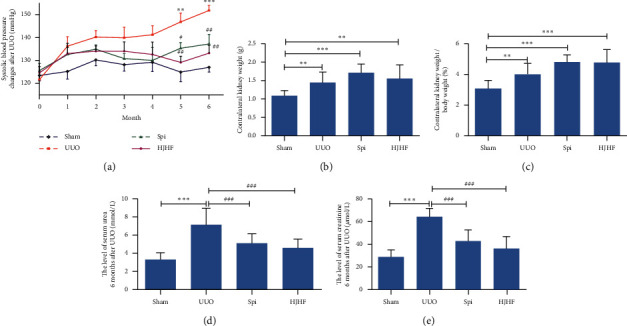
The treatment with Huayu Jiedu formula and spironolactone, respectively, improved kidney functions of 6-month-old UUO rats, although accompanied by increased systolic blood pressure and the ratio of kidney to body weight. (a) After Huoxue Jiedu Huayu formula and spironolactone intervention separately, systolic blood pressure declined 5 and 6 months after UUO compared with UUO group. vs. Sham group, ^*∗∗*^*p* < 0.01, ^*∗∗∗*^*p* < 0.001. vs. UUO group, ^#^*P* *<* 0.05, ^##^*P* *<* 0.01. (b, c) Kidney weight and the ratio of kidney to body weight increased in three groups but not in Sham group. ^*∗∗*^*p* < 0.01, ^*∗∗∗*^*p* < 0.001. (d, e) Compared with the UUO group, the levels of serum creatinine and urea went down in both Huoxue Jiedu Huayu and spironolactone group. ^*∗∗∗*^*p* < 0.001, ^###^*P* *<* 0.001.

The line chart in Figure 2 shows the changes in systolic blood pressure from 1 month to 6 months after surgery in the Sham, UUO, Spi, and HJHF groups. Compared with the UUO group, the systolic blood pressure declined at 5 and 6 months in the HJHF and Spi groups, respectively (Figure 2(a)). As shown in Figures 2(b) and 2(c), compared with the Sham group, the kidney weight and the ratio of the contralateral kidney to body weight were significantly increased in the UUO, Spi, and HJHF groups. Compared with the UUO group, serum creatinine and urea declined in both the HJHF and Spi groups (*P* *<* 0.001) (Figures 2(d) and 2(e)).

### 3.3. Inflammatory Lesions and Fibrosis of the Contralateral Kidneys Were Alleviated by Intervention with Huoxue Jiedu Huayu Formula and Spironolactone

**Figure 3:**
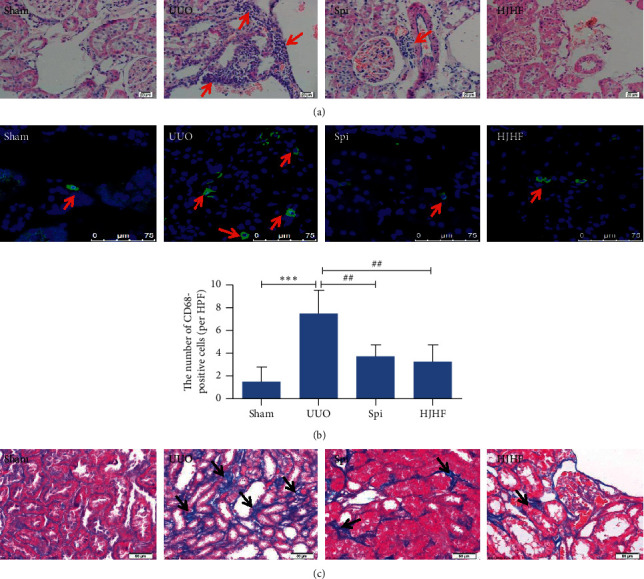
Huoxue Jiedu Huayu formula and spironolactone alleviated inflammatory lesions and fibrosis in the contralateral kidneys of 6-month-old UUO rats. (a) Inflammatory cell infiltration was observed in Sham group by HE staining, which was inhibited in Huoxue Jiedu Huayu formula and spironolactone groups. (b) Macrophages labeled with CD68 were observed by immunofluorescent staining, which were quantitatively analyzed by histogram. Macrophages are shown in green. ^*∗∗∗*^*p* < 0.001, ^##^*P* *<* 0.01. (c) Masson stain shows collagen deposition, which is in blue. Compared with the UUO group, collagen deposition observably reduced in Huoxue Jiedu Huayu formula and spironolactone groups.

As displayed by the HE staining results in Figure 3, a mass of inflammatory cells gathered around the blood vessels, and some kidney tubules were atrophied in the UUO group, while in the HJHF and Spi groups, a few inflammatory cells appeared, and the structures of the glomeruli and tubules were also essentially normal (Figure 3(a)). In addition, fluorescence staining showed that compared with the UUO group, CD68, which is a marker of macrophages in the HJHF and Spi groups, was significantly reduced (Figure 3(b)) (*P* *<* 0.01). The Masson stain shown in Figure 3(c) reveals collagen deposition in the kidney: except for blood vessels, there was a small amount of collagen at the tubular basement membrane in rats in the Sham group. However, the area of collagen stained blue was significantly increased in the UUO group and was observably reduced in both the HJHF and Spi groups.

### 3.4. MR Activation Detected by Immunohistochemistry Was Inhibited by Huoxue Jiedu Huayu Formula and Spironolactone

**Figure 4:**
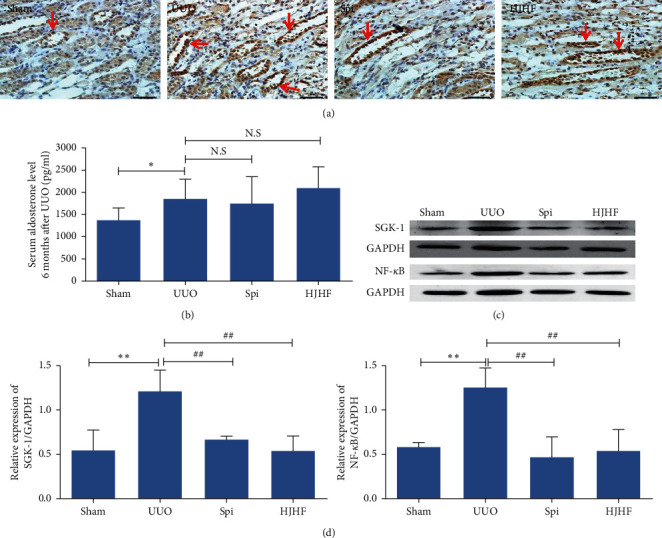
Mineralocorticoid receptor activation was inhibited by Huayu Jiedu formula and spironolactone, respectively, in the contralateral kidneys of 6-month-old UUO rats, although aldosterone levels did not change significantly compared with the UUO group. (a) The expression of NR3C2 was shown by immunohistochemistry. Compared with the UUO group, NR3C2 expression was markedly reduced in Huoxue Jiedu Huayu formula and spironolactone groups (scale bar, 50 *μ*m). (b) Histogram displayed that aldosterone levels elevated except in the Sham group. ^*∗*^*p* < 0.05. (c, d) The expression of SGK-1 and NF-*κ*B was assayed by western blotting, and protein bands were quantitatively analyzed by histogram. The SGK-1/NF-*κ*B signal pathway which indicated mineralocorticoid receptor activation was downregulated obviously after Huoxue Jiedu Huayu formula and spironolactone treatment. Data are expressed as the mean ± SD (*n* = 3). ^*∗∗*^*p* < 0.01, ^##^*P* *<* 0.01.

Compared with the Sham group, aldosterone levels increased in all, but there was no significant difference among the three groups (Figure 4(b)). NR3C2, which belongs to the MR family, translocates to the nucleus after activation [22]. As shown in the immunohistochemistry photograph, the nuclei of some kidney tubular epithelial cells appeared brown in the UUO group, but the number of brown nuclei decreased in the HJHF and Spi groups (Figure 4(a)). SGK-1 and NF-*κ*B are downstream proteins of MR activation. Western blotting showed that compared to the UUO group, SGK-1 and NF-*κ*B were obviously downregulated in the HJHF and Spi groups (*P* *<* 0.01) (Figures 4(c) and 4(d)).

### 3.5. TUNEL-Positive Cells Decreased following Huoxue Jiedu Huayu Formula and Spironolactone Treatment

**Figure 5:**
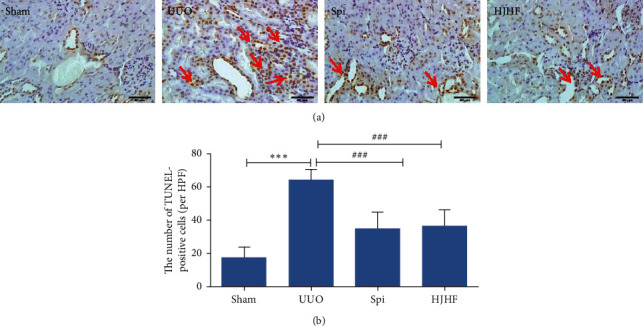
The treatment with Huoxue Jiedu Huayu formula and spironolactone, respectively, depressed DNA damage in the contralateral kidneys of 6-month-old UUO rats, which was an important feature of pyroptosis. (a) DNA damage was evaluated by TUNEL. The brown nuclei were the sites of positive expression. (b) TUNEL-positive cells were quantified by histogram. ^*∗∗∗*^*p* < 0.001, ^###^*P* *<* 0.001.

TUNEL-positive cells indicated that DNA damage was present during apoptosis or pyroptosis. TUNEL-positive cells are shown as brown nuclei. In the Sham group, very few TUNEL-positive cells were observed. Many positive cells were distributed in kidney tubules and collecting tubules in the UUO group. However, the number of positive cells in the HJHF and Spi groups was significantly lower than that in the UUO group (*P* *<* 0.001) (Figure 5).

### 3.6. The NLRP3/Caspase-1/IL-1*β* Signaling Pathway of Pyroptosis Was Obviously Inhibited by Huoxue Jiedu Huayu Formula and Spironolactone

**Figure 6:**
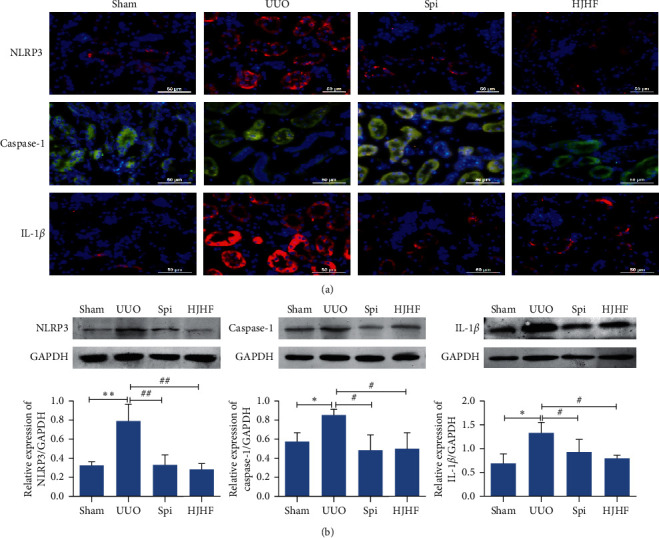
Intervention by Huoxue Jiedu Huayu formula and spironolactone obviously inhibited the NLRP3/caspase-1/IL-1*β* signal pathway of pyroptosis in the contralateral kidneys of 6-month-old UUO rats. (a) The protein expressions of NLRP3, caspase-1, and IL-1*β* were shown by immunofluorescence. NLRP3 and IL-1*β* are displayed in red, while caspase-1 is displayed in green. (b) Western blotting, respectively, demonstrate the expression of NLRP3, caspase-1, and IL-1*β*. Protein expression was quantitatively analyzed by histogram. Data are expressed as the mean ± SD (*n* = 3). ^*∗*^*p* < 0.05, ^*∗∗*^*p* < 0.01, ^#^*P* *<* 0.05, ^##^*P* *<* 0.01.

The protein expression levels of NLRP3, caspase-1, and IL-1*β* were demonstrated by immunofluorescence and Western blotting. As shown in Figure 6(a), NLRP3, caspase-1, and IL-1*β* were mainly distributed in the cytoplasm of the kidney tubular and collecting duct epithelial cells. Both NLRP3 and IL-1*β* were labeled with green fluorescence, while caspase-1 was labeled with red fluorescence. Compared with the UUO group, the expression levels of the above three proteins were downregulated significantly in the HJHF and Spi groups. Three protein bands and their quantitative analysis are displayed in Figure 6(b). Consistent with the immunofluorescence results, the expression levels of three proteins in the HJHF and Spi groups were lower than those in the UUO group.

## 4. Discussion

Obstructive nephropathy resulting from unilateral kidney or ureter obstruction could develop into CKD or kidney failure. Several studies have begun to focus on the contralateral kidney of the obstruction [3, 5]. There are different opinions on the mechanisms of contralateral kidney injury, especially regarding high perfusion, high blood pressure, and the RAAS system [23]. In previous experiments involving 10-day UUO rats, we found that high serum aldosterone levels were associated with cell pyroptosis and fibrosis of contralateral kidneys [5, 20]. However, the development of obstructive nephropathy is a long-term process, and further research is needed to determine whether these factors are involved. Therefore, we used 6-month-old UUO rats to study the relationship between aldosterone-induced pyroptosis and contralateral kidney damage and to explore the mechanism by which MRBs and HJHF protect the contralateral kidneys.

There were high pressure and high perfusion in the contralateral kidneys of UUO. In this experiment, 6-month-old unilateral nephrectomy rats were designed to observe whether high pressure and perfusion damaged the contralateral kidneys. For unilateral nephrectomy rats, we found no significant changes in kidney function and kidney histomorphology except for an increase in the ratio of the contralateral kidneys to body weight and hypertrophy of some nephrons. Serum aldosterone levels were also normal. Therefore, the above results confirmed that contralateral kidney damage may not be caused by high pressure and high perfusion in long-term obstructive nephropathy.

Inflammation triggers and promotes the development of CKD or obstructive nephropathy, such as IL-1*β*, MCP-1, and TNF-*α* [24]. Furthermore, aldosterone induces inflammatory macrophage infiltration and promotes renal fibrosis [25]. In the present study, we found that the kidney function of 6-month-old UUO rats declined, and blood pressure increased. Simultaneously, inflammatory cell infiltration, including macrophage infiltration and collagen deposition in contralateral kidneys, was observed. This is consistent with the previous study results from 10-day UUO rats [5]. Although blood pressure increased in the last two months of this experiment, hyaline changes in kidney arterioles were not observed. These findings suggest that damage to contralateral kidneys is more closely related to inflammation than blood pressure.

In recent years, the inflammatory effect of aldosterone has attracted much attention in the pathological injury of organs [25–27]. MR is the key receptor for the action of aldosterone [28]. Aldosterone binds to the MR in the cytoplasm, and is then transferred to the nucleus for the transcription of specific proteins [22]. SGK-1 and NF-кB are important downstream factors of aldosterone-MR [29], which can induce the production of inflammatory cytokines, such as MCP-1, NLRP3, pro-caspase-1, pro-IL-1*β*, and IL-6 [30, 31]. Animal studies have shown that partial nephrectomy or UUO can increase the concentration of serum aldosterone in rats, while kidney injury can be alleviated by MRB. In this study, not only serum aldosterone levels but also the number of activated NR3C2 and SGK-1/NF-кB in the contralateral kidneys were significantly upregulated in 6-month-old UUO rats. However, instead of serum aldosterone levels, the MR/SGK-1/NF-кB pathway was inhibited by MRB and HJHF.

Pyroptosis can be triggered by NLRP3 inflammasome activation, which is involved in kidney injury [31]. It has been reported that aldosterone activates the NLRP3 inflammasome, resulting in the upregulation of NLRP3 inflammasome-associated proteins, such as NLRP3, pro-caspase-1, and pro-IL-1*β*. These proteins were assembled when perceived pathogen-associated molecular patterns (PAMPs) or damage-associated molecular patterns (DAMPs) were observed [32]. Next, activated NLRP3 inflammasomes cleave GSDMD protein and hydrolyze pro-caspase-1 and pro-IL-1*β*, causing membrane perforation and the release of these inflammatory cytokines. Therefore, pyrolysis is a type of cell death characterized by an intense inflammatory response [33] and DNA damage. NLRP3/caspase-1/IL-1*β* is an important pathway for pyroptosis [34]. In this study, DNA damage was detected by using the TUNEL method, and the protein expression levels of NLRP3, caspase-1, and IL-1*β* were upregulated in the kidneys contralateral to UUO rats. In contrast, cell pyroptosis was reduced after MRB and HJHF intervention. The above results further confirmed that MR mediated pyroptosis of the contralateral kidneys of 6-month-old UUO rats.

Kidney interstitial fibrosis is a defining characteristic of CKD. According to the theory of TCM, in kidney collateral stasis, stasis can become toxic, and kidney injury is an important pathogenesis of this process. HJHF was a target for invigorating circulation and detoxification. Clinical data showed that HJHF could improve kidney function and reduce urinary protein in patients with CKD. In the experiment, we also observed that collagen deposition decreased and kidney function improved in the HJHF group. Compared with the UUO group, serum aldosterone levels were not significantly altered, but the amount of activated NR3C2 and pyroptosis was significantly reduced in the HJHF group, suggesting that HJHF prevented aldosterone-induced pyroptosis by inhibiting MR activation, thereby protecting contralateral kidneys. Nevertheless, the treatment strategies of HJHF emphasize the holistic view; in this study, we have only explored it from the perspective of MR and pyroptosis. Thus, the detailed mechanism needs to be further studied.

## 5. Conclusion

This study confirms that excessive activation of aldosterone-MR leads to cell pyroptosis and contralateral kidney injury in 6-month-old UUO rats. Furthermore, HJHF and MRB reduced cell pyroptosis through the NLRP3/caspase-1/IL-1*β* pathway. Intervention by MRBs at an early stage may be a new treatment strategy for reducing the progression of chronic obstructive nephropathy.

## Data Availability

The data used to support the findings of this study are available from the corresponding author upon request.
